# Contemporary Perspectives on Balloon Rupture in Calcified Lesions

**DOI:** 10.1016/j.jaccas.2026.108614

**Published:** 2026-06-10

**Authors:** Raymond Chi-Yan Fung

**Affiliations:** Cardiac Catheterization Laboratories, Princess Margaret Hospital, Hong Kong SAR, China

**Keywords:** complication, intravascular ultrasound, imaging, percutaneous coronary intervention

**Figure:**
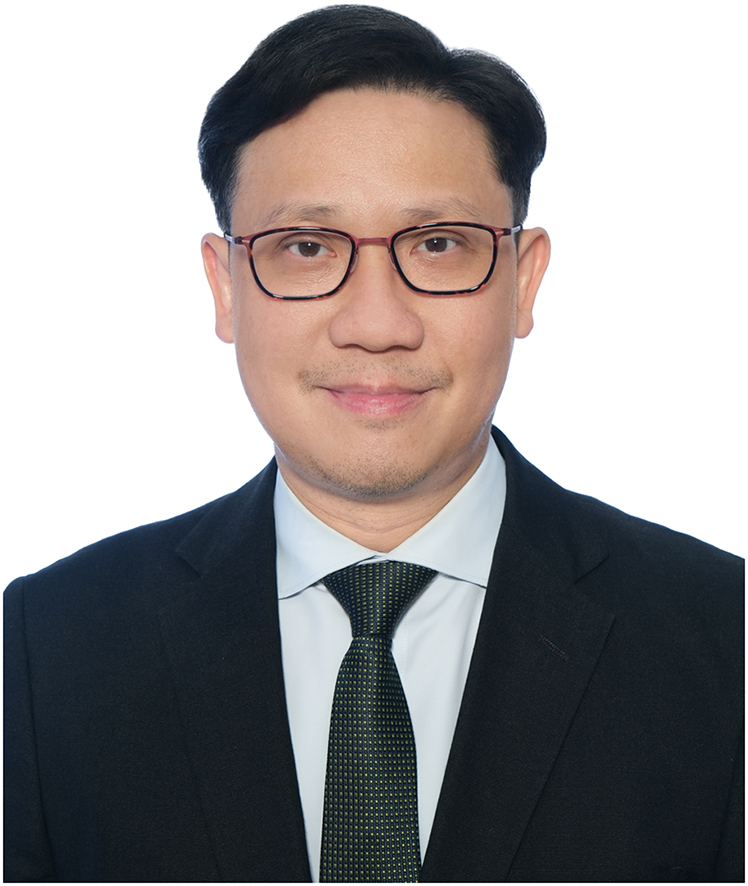

Coronary intervention in calcified lesions is challenging. The presence of calcium in coronary lesions can hinder the passage of interventional devices, create lesions that are resistant to balloon dilation, and lead to underexpanded stents. These challenges contribute to lower procedural success rates and higher complication rates, including balloon rupture and coronary dissection. In this issue of *JACC: Case Reports*, Nakata et al^1^ describe a case of extensive retrograde dissection after intravascular lithotripsy (IVL) balloon rupture. Their report underscores the need to integrate diagnostic imaging with knowledge of device and lesion characteristics for optimal outcomes.

Balloon rupture may lead to severe issues, including coronary perforation, dissection, entrapment of balloon materials causing obstruction, myocardial ischemia, cardiogenic shock, malignant arrhythmias, and cardiac arrest.^2^, ^3^, ^4^ Typical patterns of conventional balloon rupture include pinhole, circumferential, and longitudinal rupture. Vessel tortuosity and angulation, as well as sharp and protruding eccentric calcification increase the risk of pinhole perforation by puncturing balloon materials. Procedurally, balloon rupture risk increases with oversizing and repeated reuse because of material fatigue.^5^ Inflating a balloon beyond its rated burst pressure and subsequently attempting rapid retrieval without adequate deflation significantly increases the risk of entrapment due to circumferential balloon rupture.^6^ The “dog-bone” effect seen in severely concentric calcified lesions cause the more compliant ends of the balloon to expand, generating significant tension at the waist. This tension can result in difficulty or delay when deflating the distal part of the balloon. If the balloon is pulled back while still partially inflated, the mechanical force against the rigid calcium may physically tear the balloon circumferentially.^6^

Tears may also result from design issues related to the materials used in manufacturing the balloon.^5^ Semicompliant balloons, composed of polyether block amide (PEBA/Pebax) or nylon (commonly, Nylon-12), demonstrate a higher likelihood of rupture when compared with noncompliant balloons, which are typically manufactured from polyethylene terephthalate (PET) or high-durometer nylon.^7^ Both PEBA and Nylon-12 balloons possess greater radial tensile strength relative to longitudinal tensile strength. This characteristic is more pronounced in Nylon-12 than in PEBA, resulting in Nylon-12 balloons exhibiting stronger axial orientation and an increased propensity for rupturing along the longitudinal axis.^5^ PET, on the other hand, is rigid and susceptible to pinhole ruptures.^8^

The clinical effect of balloon rupture is largely dictated by the type of rupture. A pinhole tear creates a high-velocity jet of contrast that may lead to coronary dissection, intramural hematoma, or perforation.^2^^,^^9^ The case described by Nakata et al^1^ underscores how coronary anatomy influences both the direction and the extent of hematoma propagation. They proposed that anatomical angulation may “steer” the trajectory of the hematoma, whereas thinning or scarring of the medial layer behind calcified plaque may restrict further intramural extension. Circumferential tearing can cause balloon detachment and subsequent embolization, resulting in coronary obstruction.^10^ In contrast, longitudinal tearing is considered the most benign type and rarely causes serious complications.^2^

The Shockwave IVL balloon (Shockwave Medical) is equipped with emitters that generate shock waves inside the balloon, which are used to break up tough, calcified lesions within the vessel wall. Only a low inflation pressure (4 atm) is required to achieve such lesion dissection, potentially resulting in less medial injury. Although an IVL balloon has a design different from that of conventional balloons, complications related to IVL balloon rupture have also been reported.^11^ One case report described tears occurring near the emitters, thought to result from thermal injury caused by nonuniform balloon expansion in the setting of eccentric calcification. In contrast, Nakata et al^1^ postulated that the dissection arose from a pinhole rupture produced by focal intimal injury due to stress concentrated by eccentric calcium. Because of variations in design, different coronary balloons exhibit distinct rupture mechanisms. By recognizing these differences in both their construction and failure modes, clinicians can more effectively assess the risks and likely outcomes associated with each device.

The use of intracoronary imaging is crucial for complex calcified lesions intervention to ensure selection of the most suitable devices, including balloons or atherectomy instruments. Optical coherence tomography (OCT) and intravascular ultrasound (IVUS) offer complementary capabilities during coronary interventions. OCT more precisely assesses calcium distribution, including its length, burden, eccentricity, and calcified nodules. Additionally, guidewire bias and vessel angulation can be seen. This additional information can help to prevent balloon rupture. Selecting appropriately sized balloons can help prevent oversizing. Treating eccentric calcified lesions, particularly calcified nodules, remains challenging, as currently available calcium modification devices—including specially designed balloons and atherectomy tools—are not ideal. For certain calcified lesions with a favorable guidewire bias, atherectomy devices may be appropriate.^12^ In other cases, IVL can be considered, but caution is needed given the potential risk of balloon rupture. Besides preprocedural planning, Nakata et al^1^ illustrate the distinct value each imaging modality provides in elucidating the pathophysiology that facilitates both detection and management of the complication. If balloon rupture is suspected, IVUS is preferable because OCT requires high-pressure contrast injection, which could worsen dissection or perforation. The extensive hematoma propagation in Nakata et al^1^ may result from a vulnerable entry due to the hydraulic stress from contrast injection. IVUS offers a safer alternative in this situation. It can demonstrate dissection flaps and delineate the extent of dissection or intramural hematoma without requiring additional contrast administration. This information is essential for planning further management.

## Funding Support and Author Disclosures

The author has reported no relationships relevant to the contents of this paper to disclose.
